# Genomic Surveillance of SARS-CoV-2 Viruses Collected during the Ending Phase of the First Wave of the COVID-19 Pandemic in Bangladesh

**DOI:** 10.1128/MRA.00496-21

**Published:** 2021-07-22

**Authors:** Munira Jahan, Arittra Bhattacharjee, Raad Rahmat, S. M. Rashedul Islam, Tahmina Akhter, Ishtiaque Ahammad, Mohammad Uzzal Hossain, Saif Ullah Munshi, M. Salimullah

**Affiliations:** a Department of Virology, Bangabandhu Sheikh Mujib Medical University, Shahbag, Dhaka, Bangladesh; b Bioinformatics Division, National Institute of Biotechnology, Ganakbari, Ashulia, Savar, Dhaka, Bangladesh; c Biotechnology Program, Department of Mathematics and Natural Sciences, School of Data and Sciences, BRAC University, Mohakhali, Dhaka, Bangladesh; d Molecular Biotechnology Division, National Institute of Biotechnology, Ganakbari, Ashulia, Savar, Dhaka, Bangladesh; DOE Joint Genome Institute

## Abstract

Mutations, deletions, and the emergence of new variants of severe acute respiratory syndrome coronavirus 2 (SARS-CoV-2) may pose a serious health threat. Here, we report the genome sequences of SARS-CoV-2 viruses that were collected from SARS-CoV-2-infected patients during the end phase of the first wave of the COVID-19 pandemic in Dhaka, Bangladesh.

## ANNOUNCEMENT

Severe acute respiratory syndrome coronavirus 2 (SARS-CoV-2) (family *Coronaviridae*, genus *Betacoronavirus*) is the causative agent of coronavirus disease 2019 (COVID-19). New variants of SARS-CoV-2 were detected as part of the second wave of the COVID-19 pandemic starting in March 2021 (https://www.worldometers.info/coronavirus/country/bangladesh/). Between November 2020 and February 2021, the rate of COVID-19 cases started to decrease and remained relatively low in Bangladesh. To analyze the types of SARS-CoV-2 variants which were circulating during the end phase of the first wave, nasopharyngeal swabs from SARS-CoV-2-infected patients were collected from the Department of Virology, Bangabandhu Sheikh Mujib Medical University (BSMMU), during November and December 2020. (International, national, and/or institutional guidelines were followed according to the Institutional Review Board [IRB] of BSMMU; the ethical approval number is BSMMU/2020/6320.) Nasopharyngeal swabs were tested for SARS-CoV-2 RNA via real-time reverse transcription-PCR (RT-PCR), and 23 RT-PCR-positive samples (cycle threshold [Ct], ≤25) were selected for the study.

Viral RNA was extracted from the nasopharyngeal samples using the QIAamp viral minikit (Qiagen; catalog number 52904). cDNA synthesis and library preparation were performed using the Illumina RNA prep kit with enrichment (catalog number 20040537) and IDT for Illumina DNA/RNA UD indexes (catalog number 20027213), followed by enrichment with the Respiratory Virus Oligos Panel v2 (Illumina; catalog number 20044311). All procedures were conducted according to the Illumina RNA prep with enrichment (L) tagmentation reference guide (1000000124435 v03). Sequencing was performed on the Illumina MiniSeq system. The genomes were then processed using the DRAGEN RNA pathogen detection app. The low-quality raw reads were trimmed using Trimmomatic v0.36, and the *de novo* genome assembly was performed using SPAdes v03. These steps were executed using default parameters (1, 2). In total, 23 contigs were obtained and compared with the SARS-CoV-2 isolate Wuhan-Hu-1 (GenBank accession number NC_045512.2); the genome coverage was 99.86% to 99.93%.

**FIG 1:**
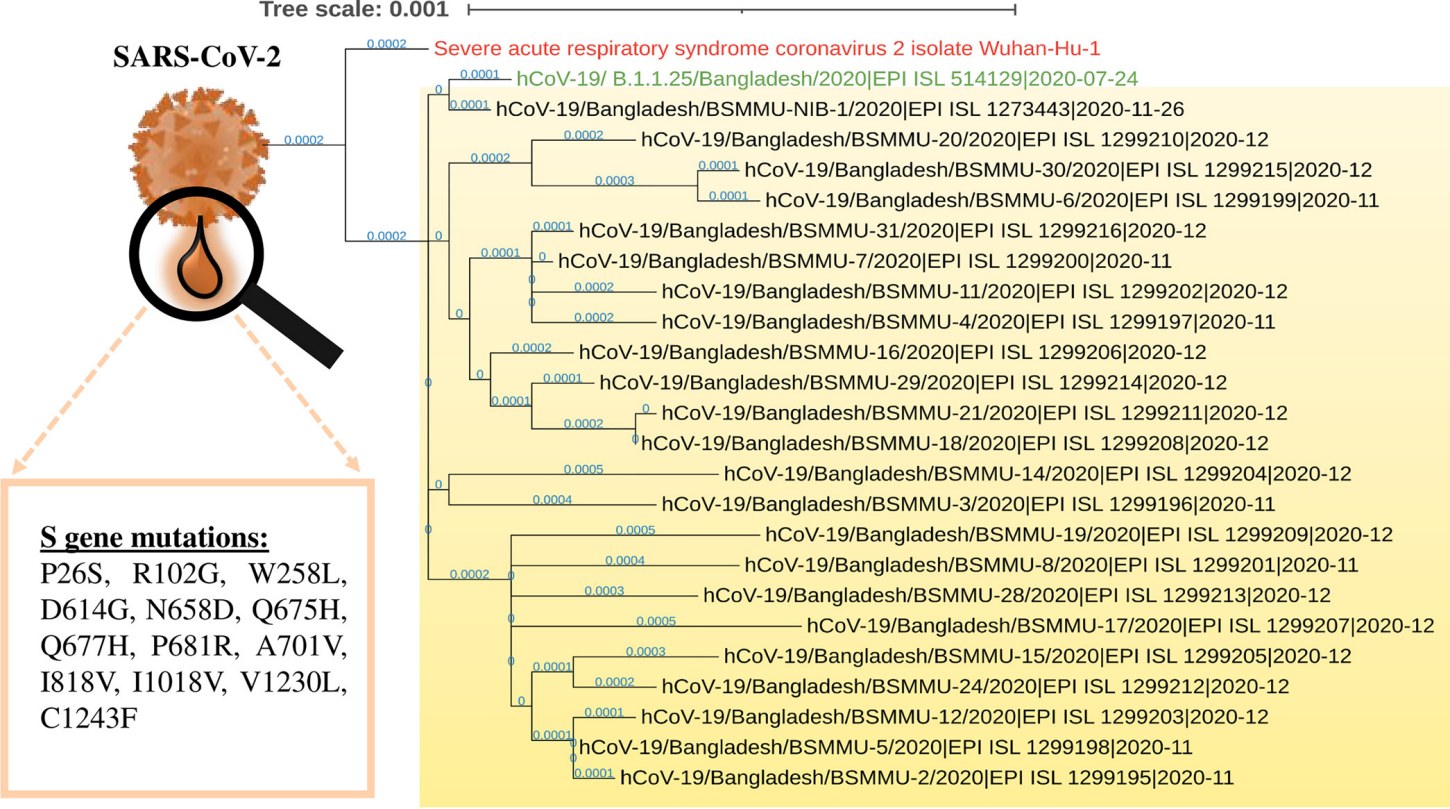
Phylogenetic analysis of the collected SARS-CoV-2 viruses. A tree was constructed using the approximately maximum-likelihood method with MAFFT nucleotide alignment and the generalized time-reversible model (GTR) + CAT nucleotide evolution model. A scale bar with 0.001 branch length is given at the top. The collected SARS-CoV-2 viruses were from the B.1.1.25 lineage of the GR clade (inside the pale yellow box).

A phylogenetic tree was constructed as previously described (3) (Fig. 1). In short, the FASTA files of the assembled genome sequences were aligned using MAFFT (keeping the parameters unchanged), and the tree was created using FastTree v2.1.10 (4, 5) via the Galaxy platform (6). Here, SARS-CoV-2 isolate Wuhan-Hu-1 was used as the reference genome, and another lineage B.1.1.25 variant (hCoV-19/Bangladesh/BCSIR-NILMRC-356/2020|EPI_ISL_514129|2020-07-24) was added for comparison. The phylogenetic tree was visualized using iTOL (7). All mutations were calculated using CoV-GLUE (8), with SARS-CoV-2 isolate Wuhan-Hu-1 used as the reference genome (Table 1).

**TABLE 1:** Single nucleotide polymorphisms and mutations in proteins that were detected in the collected SARS-CoV-2 viruses

Virus name	SNPs^*a*^	No. of SNPs^*a*^	Genes and mutations	SRA accession no.^*b*^
hCoV-19/Bangladesh/BSMMU-NIB-1/2020|EPI_ISL_1273443|2020-11-26	A1163T, C3037T, A11451G, C14408T, C22388T, A23403G, C28253T, G28881A, G28882A, G28883C	10	nsp2: I120F; nsp6: Q160R; nsp12: P323L; S: D614G; N: R203K; N: G204R	SRX11079871
hCoV-19/Bangladesh/BSMMU-2/2020|EPI_ISL_1299195|2020-11	A1163T, C2094T, C3037T, C10594T, C14408T, C14925T, G20238T, A23403G, C23664T, C25587T, C25904T, G26211T, G27327T, G28881A, G28882A, G28883C	16	nsp2: I120F; nsp2: S430L; nsp12: P323L; nsp15: R206S; S: D614G; S: A701V; ORF3a: S171L; ORF6: K42N; N: R203K; N: G204R	SRX11079872
hCoV-19/Bangladesh/BSMMU-3/2020|EPI_ISL_1299196|2020-11	C583T, A1163T, C3037T, C10582T, C13923T, C14408T, C16260T, G20352T, A21866G, A23403G, C25831T, C26060T, A26189G, G27777T, G28881A, G28882A, G28883C	17	nsp2: I120F; nsp12: P323L; nsp15: Q244H; S: R102G; S: D614G; ORF3a: L147F; ORF3a: T223I; ORF3a: E266G; ORF7b: D8Y; N: R203K; N: G204R	SRX11079883
hCoV-19/Bangladesh/BSMMU-4/2020|EPI_ISL_1299197|2020-11	C335T, G434A, A1163T, C3037T, A3652G, T4861C, A9436G, C14408T, C15049T, A16550G, A23403G, C23604G, T27207C, G27505C, C27577T, G28881A, G28882A, G28883C	18	nsp1: R24C; nsp1: E57K; nsp2: I120F; nsp12: P323L; nsp12: P537S; nsp13: D105G; S: D614G; S: P681R; ORF7a: G38R; N: R203K; N: G204R	SRX11079886
hCoV-19/Bangladesh/BSMMU-5/2020|EPI_ISL_1299198|2020-11	A1163T, C2094T, C3037T, C10594T, C14408T, G20238T, A23403G, C23664T, C25904T, G26211T, G27327T, G28881A, G28882A, G28883C	14	nsp2: I120F; nsp2: S430L; nsp12: P323L; nsp15: R206S; S: D614G; S: A701V; ORF3a: S171L; ORF6: K42N; N: R203K; N: G204R	SRX11079887
hCoV-19/Bangladesh/BSMMU-6/2020|EPI_ISL_1299199|2020-11	A1163T, C3037T, G3955T, C5392T, A6063G, A8412G, C9611T, G11083T, C14408T, A16790T, C19602T, A23403G, C23604G, C25782T, T26604C, C26681T, C26996T, A27379G, G28881A, G28882A, G28883C, G29402T	22	nsp2: I120F; nsp3: K412N; nsp3: D1115G; nsp3: K1898R; nsp4: L353F; nsp6: L37F; nsp12: P323L; nsp13: Y185F; S: D614G; S: P681R; M: F28L; ORF6: I60V; N: R203K; N: G204R; N: D377Y	SRX11079888
hCoV-19/Bangladesh/BSMMU-7/2020|EPI_ISL_1299200|2020-11	C335T, G434A, A1163T, C3037T, A3652G, A9436G, C14408T, A23403G, C23604G, A24614G, G28881A, G28882A, G28883C	13	nsp1: R24C; nsp1: E57K; nsp2: I120F; nsp12: P323L; S: D614G; S: P681R; S: I1018V; N: R203K; N: G204R	SRX11079889
hCoV-19/Bangladesh/BSMMU-8/2020|EPI_ISL_1299201|2020-11	A1163T, C2094T, C3037T, T4149G, T6238C, C7423T, G7427T, C7834T, T8761C, C14408T, G20238T, A21469C, A23403G, C25844T, C25904T, G26211T, C27389T, G28881A, G28882A, G28883C, T29029C, A29594G	22	nsp2: I120F; nsp2: S430L; nsp3: V477G; nsp3: A1570S; nsp12: P323L; nsp15: R206S; nsp16: I271L; S: D614G; ORF3a: T151I; ORF3a: S171L; N: R203K; N: G204R; ORF10: I13V	SRX11079890
hCoV-19/Bangladesh/BSMMU-11/2020|EPI_ISL_1299202|2020-12	C335T, G434A, C874T, A1163T, A1866G, C3037T, A3652G, A9436G, C14408T, G20679T, A23403G, C23604G, G25621T, C27741T, G28881A, G28882A, G28883C, C29541T	18	nsp1: R24C; nsp1: E57K; nsp2: I120F; nsp2: Y354C; nsp12: P323L; S: D614G; S: P681R; ORF3a: V77F; N: R203K; N: G204R	SRX11079891
hCoV-19/Bangladesh/BSMMU-12/2020|EPI_ISL_1299203|2020-12	A1163T, C2094T, C2459T, C3037T, C10594T, G12070T, C14408T, G20238T, A23403G, C23664T, G25290T, C25904T, G26211T, G27327T, G28881A, G28882A, G28883C	17	nsp2: I120F; nsp2: S430L; nsp2: P552S; nsp12: P323L; nsp15: R206S; S: D614G; S: A701V; S: C1243F; ORF3a: S171L; ORF6: K42N; N: R203K; N: G204R	SRX11079892
hCoV-19/Bangladesh/BSMMU-14/2020|EPI_ISL_1299204|2020-12	A1163T, T1728C, C3037T, A3840G, C7945T, A10323G, G10618A, C12781T, C14408T, G16935T, C17676T, G20014T, C20762T, C21637T, A23403G, A23534G, C26060T, G28300T, G28881A, G28882A, G28883C	21	nsp2: I120F; nsp2: V308A; nsp3: E374G; nsp5: K90R; nsp12: P323L; nsp13: M233I; nsp15: D132Y; nsp16: T35I; S: D614G; S: N658D; ORF3a: T223I; N: Q9H; N: R203K; N: G204R	SRX11079873
hCoV-19/Bangladesh/BSMMU-15/2020|EPI_ISL_1299205|2020-12	A1163T, C2094T, C3037T, T6640C, C14408T, C17285T, G20238T, C20930T, C22987A, C23248T, A23403G, G23587C, C24382T, G25250T, C25904T, G26211T, G27327T, G28881A, G28882A, G28883C, C28905T	21	nsp2: I120F; nsp2: S430L; nsp12: P323L; nsp13: S350L; nsp15: R206S; nsp16: T91M; S: D614G; S: Q675H; S: V1230L; ORF3a: S171L; ORF6: K42N; N: R203K; N: G204R; N: A211V	SRX11079874
hCoV-19/Bangladesh/BSMMU-16/2020|EPI_ISL_1299206|2020-12	G434A, C678T, A1163T, C3037T, A13803G, C14408T, A23403G, C23604G, G28079T, C28394A, G28881A, G28882A, G28883C	13	nsp1: E57K; nsp1: A138V; nsp2: I120F; nsp12: P323L; S: D614G; S: P681R; N: R203K; N: G204R	SRX11079875
hCoV-19/Bangladesh/BSMMU-17/2020|EPI_ISL_1299207|2020-12	A1163T, C2094T, C2455T, C3037T, A3904G, C6258T, C11674T, C14220T, C14408T, C14821T, G20238T, C21638T, A23403G, A24014G, C25904T, C26013T, C26176T, G26211T, C28695T, G28881A, G28882A, G28883C, G29260C, T29317C, C29409T	25	nsp2: I120F; nsp2: S430L; nsp3: T1180I; nsp12: P323L; nsp12: P461S; nsp15: R206S; S: P26S; S: D614G; S: I818V; ORF3a: S171L; ORF3a: P262S; N: T141I; N: R203K; N: G204R; N: T379I	SRX11080822
hCoV-19/Bangladesh/BSMMU-18/2020|EPI_ISL_1299208|2020-12	G434A, A1163T, C3037T, G4720T, C8950T, T14305C, C14408T, A23403G, C23604G, C25658T, C27600T, G28079T, G28881A, G28882A, G28883C, C29272T	16	nsp1: E57K; nsp2: I120F; nsp12: Y289H; nsp12: P323L; S: D614G; S: P681R; ORF3a: T89I; N: R203K; N: G204R	SRX11079876
hCoV-19/Bangladesh/BSMMU-19/2020|EPI_ISL_1299209|2020-12	A1163T, C2094T, C2135T, C3037T, C5934T, G7791A, C9487T, C14408T, G20134T, G20176T, G20238T, T22204G, A23403G, C25904T, G25947T, G26211T, C26801T, G28001T, T28118C, G28881A, G28882A, G28883C, C29520T	23	nsp2: I120F; nsp2: S430L; nsp2: L444F; nsp3: T1072I; nsp3: G1691D; nsp12: P323L; nsp15: V172L; nsp15: V186F; nsp15: R206S; S: D614G; ORF3a: S171L; ORF3a: Q185H; N: R203K; N: G204R; N: S416L	SRX11079877
hCoV-19/Bangladesh/BSMMU-20/2020|EPI_ISL_1299210|2020-12	A1163T, C3037T, G3955T, C14408T, T17400C, G22335T, A23403G, C23604G, G25617T, T26604C, C26681T, A27379G, C27434T, G28881A, G28882A, G28883C	16	nsp2: I120F; nsp3: K412N; nsp12: P323L; S: W258L; S: D614G; S: P681R; ORF3a: K75N; M: F28L; ORF6: I60V; ORF7a: T14I; N: R203K; N: G204R	SRX11079878
hCoV-19/Bangladesh/BSMMU-21/2020|EPI_ISL_1299211|2020-12	G434A, A1163T, C3037T, G4720T, C8950T, C11750T, T14305C, C14408T, A23403G, C23604G, C25658T, C27600T, G28079T, G28881A, G28882A, G28883C, C29272T	17	nsp1: E57K; nsp2: I120F; nsp6: L260F; nsp12: Y289H; nsp12: P323L; S: D614G; S: P681R; ORF3a: T89I; N: R203K; N: G204R	SRX11079879
hCoV-19/Bangladesh/BSMMU-24/2020|EPI_ISL_1299212|2020-12	A1163T, C2094T, C3037T, T6640C, C14408T, C19274T, G20238T, C21034T, A23403G, G23587C, C25904T, G26211T, G27327T, G28881A, G28882A, G28883C, G29648T	17	nsp2: I120F; nsp2: S430L; nsp12: P323L; nsp14: P412L; nsp15: R206S; nsp16: L126F; S: D614G; S: Q675H; ORF3a: S171L; ORF6: K42N; N: R203K; N: G204R; ORF10: D31Y	SRX11079880
hCoV-19/Bangladesh/BSMMU-28/2020|EPI_ISL_1299213|2020-12	A1163T, C2094T, C3037T, C6026T, C6726T, G7756T, C14408T, G20238T, G21255T, A23403G, G25489T, C25904T, G26211T, G28198C, G28209T, G28881A, G28882A, G28883C, G28908T	19	nsp2: I120F; nsp2: S430L; nsp3: P1103S; nsp3: T1336I; nsp3: K1679N; nsp12: P323L; nsp15: R206S; S: D614G; ORF3a: A33S; ORF3a: S171L; ORF8: C102S; ORF8: E106*; N: R203K; N: G204R; N: G212V	SRX11079881
hCoV-19/Bangladesh/BSMMU-29/2020|EPI_ISL_1299214|2020-12	G434A, A1163T, C3037T, C10456T, C11750T, C14408T, A23403G, G23593T, C23604G, G28079T, G28881A, G28882A, G28883C, C29272T	14	nsp1: E57K; nsp2: I120F; nsp6: L260F; nsp12: P323L; S: D614G; S: Q677H; S: P681R; N: R203K; N: G204R	SRX11079882
hCoV-19/Bangladesh/BSMMU-30/2020|EPI_ISL_1299215|2020-12	A1163T, C3037T, G3955T, C5392T, A6063G, C6145T, A8412G, C9611T, C10030T, C14408T, C19602T, A23403G, C23604G, C25782T, T26604C, C26681T, C26996T, A27379G, G28881A, G28882A, G28883C	21	nsp2: I120F; nsp3: K412N; nsp3: D1115G; nsp3: K1898R; nsp4: L353F; nsp12: P323L; S: D614G; S: P681R; M: F28L; ORF6: I60V; N: R203K	SRX11079884
hCoV-19/Bangladesh/BSMMU-31/2020|EPI_ISL_1299216|2020-12	C335T, G434A, A1163T, C3037T, A3652G, A9436G, C14408T, C19813T, A23403G, C23604G, G28881A, G28882A, G28883C	13	nsp1: R24C; nsp1: E57K; nsp2: I120F; nsp12: P323L; nsp15: P65S; S: D614G; S: P681R; N: R203K; N: G204R	SRX11079885

a SNP, single nucleotide polymorphism.

b The raw reads are available in the NCBI Sequence Read Archive (SRA), and accession numbers are given for each library.

Finally, the SARS-CoV-2 genome sequences were uploaded to the Global Initiative on Sharing All Influenza Data (GISAID) database (9) and the Pangolin COVID-19 Lineage Assigner (10). All viruses were from the B.1.1.25 lineage of the GR clade. Among them, P681R was the second most common mutation in the S gene (11 out of 23), after D614G, which has also been observed in the Indian lineages B.1.617.1, B.1.617.2, and B.1.617.3. A mutation in a similar position (P681H) was also detected in the UK variant B.1.1.7 (https://www.cdc.gov/). A mutation in P681 might affect the viral transmission and infectivity (11, 12). This lineage was predominant during the peak of the first wave in Bangladesh (13); however, in the later stages, its spike glycoprotein (S) gained new mutations (Fig. 1), which, with further changes, might help the virus escape humoral immunity in the near future and raise another surge of B.1.1.25-mediated COVID-19 cases. Therefore, continuous surveillance of this variant is necessary.

### Data availability.

The whole-genome sequences of the collected SARS-CoV-2 isolates have been deposited in GISAID (https://www.gisaid.org/) under the accession numbers EPI_ISL_1299195 to EPI_ISL_1299216 and EPI_ISL_1273443 and the GenBank accession numbers MZ148590, MZ148591, MZ148592, MZ148593, MZ148594, MZ148595, MZ148596, MZ148597, MZ148598, MZ148599, MZ148600, MZ148601, MZ148602, MZ148603, MZ148604, MZ148605, MZ148606, MZ148607, MZ148608, MZ148609, MZ148610, MZ148611, and MZ148612. Moreover, the raw data have been submitted under the NCBI BioProject accession number PRJNA735597.
